# Evidence for progressive neurodegeneration in iatrogenic cerebral amyloid angiopathy

**DOI:** 10.1002/alz.71640

**Published:** 2026-06-30

**Authors:** Larysa Panteleienko, Gargi Banerjee, Dermot Mallon, Edgar Chan, Simon Fandler‐Höfler, Rupert Oliver, Victoria Harvey, Zane Jaunmuktane, John Collinge, David J Werring

**Affiliations:** ^1^ Department of Translational Neuroscience and Stroke Stroke Research Centre, UCL Queen Square Institute of Neurology London UK; ^2^ Deptartment of Neurology Bogomolets National Medical University Kyiv Ukraine; ^3^ Institute of Prion Diseases MRC Prion Unit at UCL London UK; ^4^ National Prion Clinic, National Hospital for Neurology and Neurosurgery University College London Hospitals NHS Foundation Trust London UK; ^5^ Neuroradiological Academic Unit UCL Queen Square Institute of Neurology London UK; ^6^ Lysholm Department of Neuroradiology National Hospital for Neurology and Neurosurgery London UK; ^7^ National Hospital for Neurology and Neurosurgery University College London Hospitals NHS Foundation Trust London UK; ^8^ Department of Neurology Medical University of Graz Graz Austria; ^9^ Division of Neuropathology National Hospital for Neurology and Neurosurgery University College London Hospitals NHS Foundation Trust London UK; ^10^ Department of Clinical and Movement Neurosciences and Queen Square Brain Bank for Neurological Disorders Queen Square Institute of Neurology London UK

**Keywords:** amyloid beta, cadaveric dura mater, iatrogenic Alzheimer's disease, iatrogenic cerebral amyloid angiopathy, neurosurgery, prion

## Abstract

**INTRODUCTION:**

Iatrogenic transmission of amyloid beta can cause cerebral amyloid angiopathy (CAA) and Alzheimer's disease (AD), but the relationship between these phenotypes is unclear.

**METHODS:**

We retrospectively analyzed standardized neuropsychological and neuroimaging data from 11 patients with iatrogenic CAA (iCAA). Brain MRI was assessed for medial temporal lobe atrophy (MTA), the posterior atrophy score for parietal atrophy, and global cortical atrophy (GCA).

**RESULTS:**

All patients (mean age 42 ± 8.3 years) had childhood neurosurgery; 91% had confirmed cadaveric dura exposure. Six patients (55%) presented with intracerebral hemorrhage, and none showed MTA, parietal atrophy, or GCA at presentation. Over a median 5‐year follow‐up, 8/11 (73%) developed atrophy on at least one score, moderate to severe in three patients. Cognitive impairment was present in 9/11 (82%) at a median 3‐year follow‐up. AD was confirmed histopathologically in 2/4 (50%) examined cases.

**DISCUSSION:**

Progressive brain atrophy and cognitive impairment are common in iCAA, suggesting frequent co‐existing neurodegeneration and possible AD pathology. Vigilance for cognitive decline may enable earlier identification and management.

## Highlights

- In a retrospectively analyzed cohort of 11 patients with iatrogenic CAA, progressive neurodegeneration (i.e. medial temporal, parietal, or global cortical atrophy), developed in 73% over a median observation period of 5 years.
- Cognitive impairment was common in iatrogenic CAA, affecting 82% of cases, with 22% major and 78% minor impairment at a median follow‐up period of 3 years.
- Cognitive and imaging findings suggest progressive neurodegeneration with at least some contribution from co‐existing Alzheimer's pathology, either independent of, or consequent to, iCAA.

## 1 INTRODUCTION

Iatrogenic cerebral amyloid angiopathy (iCAA) is caused by the transmission of amyloid beta (Aβ) pathology during medical procedures^1^, ^2^; the majority of reported cases are consequent to procedures involving cadaveric dura mater.^3^, ^4^ Clinical presentation typically occurs several decades later and resembles sporadic CAA, with nearly all reported patients developing intracerebral hemorrhage (ICH) during the course of their illness.^4^, ^5^ More recently, cases of iatrogenic Alzheimer's disease following treatment with cadaveric human growth hormone have been described.^6^, ^7^ This suggests that iatrogenic transmission of Aβ pathology can result in both vasculopathic (CAA) and neurodegenerative (Alzheimer's disease) phenotypes, but the extent to which these phenotypes overlap or interact is unknown.

To address this question, we retrospectively reviewed whether patients with iCAA had evidence of cognitive impairment or radiological changes suggestive of neurodegeneration (atrophy).

## 2 METHODS

### 2.1 Study design and participants

We performed a retrospective review of all patients with iCAA identified and referred to our quaternary center between 2015 and 2025. The entire cohort (*n* = 11) was evaluated in our specialist intracranial hemorrhage clinic. Each visit included a same‐day neurological assessment, formal neuropsychometry, and a magnetic resonance imaging (MRI) brain with a standardized cerebrovascular protocol.

### 2.2 Neuropsychometry

Neuropsychometry was performed using a standardized battery of tests (Supplementary Information).^8^ Test performance was scored according to published normative data. Impairment in verbal and performance IQ was defined as a difference ≥10 points between the patient's actual performance and their estimated premorbid functioning based on the National Adult Reading Test (NART). Impairment in a focal cognitive domain was classed as scoring at or below the fifth percentile on any one test within the domain, except for the executive domain, where failure on two or more tests was required or only one if it was a screening measure (Table S1). Minor and major cognitive disorders were defined in accordance with the *Diagnostic and Statistical Manual of Mental Disorders*, Fifth Edition.^9^ Minor is defined as an impairment in one or more cognitive domains that necessitates increased effort, compensatory strategies, or external accommodations but does not interfere with independence in activities of daily living; major is defined as a multidomain impairment that interferes with independent daily function.

### 2.3 Neuroimaging evaluation of neurodegeneration

MRI scans were reviewed by a consultant neurologist with training in neuroimaging evaluation (LP) and an experienced consultant neuroradiologist (DM). Medial temporal atrophy (MTA) was rated using the standardized Medial Temporal Atrophy score (range: 0 to 4) on coronal T1 sequences.^10^ For patients younger than 65 years, MTA scores ≥1 were considered abnormal.

The posterior atrophy score was rated using the Koedam score (score 0 to 3) using axial, coronal, and sagittal T1 or Fluid‐attenuated inversion recovery (FLAIR) sequences.^11^ Global cortical atrophy (GCA) was rated using the Global Cortical Atrophy Scale (score 0 to 3) using axial T2 (inverted for assessment) or FLAIR sequences.^12^ For all scores, ≥2 was considered as moderate to severe atrophy.

Inter‐rater reliability was assessed using Cohen's kappa statistic. Agreement between raters was high, with *κ* = 0.83 (93.9% agreement) for MTA, *κ* = 0.86 (95.5% agreement) for the Koedam score, and *κ* = 0.66 (86.4% agreement) for GCA.

### 2.4 Consent statement

This report follows the Consensus‐based Clinical Case Reporting (CARE) and Strengthening the Reporting of Observational Studies in Epidemiology (STROBE) reporting guidelines. Written consent for publication was obtained for all living patients or their next of kin.

**RESEARCH IN CONTEXT**

1. **Systematic review**: The authors reviewed recent high‐profile publications demonstrating that Aβ pathology can be transmitted through medical exposure. Multiple studies describe iCAA, which often presents decades later as early‐onset intracerebral haemorrhage. More recently, cases of iatrogenic Alzheimer's disease following treatment with cadaveric human growth hormone have been reported, expanding the spectrum of iatrogenic Aβ transmission beyond purely vascular pathology.
2. **Interpretation**: In this observational cohort study of 11 patients, nearly three‐quarters developed progressive brain atrophy over a median follow‐up of 5 years, highlighting a potential role for neurodegeneration in iCAA. Alzheimer's disease–related histopathological features were identified in half of the examined cases (*n* = 4), suggesting that Alzheimer's co‐pathology may contribute to disease mechanisms and progression in at least a subset of individuals.
3. **Future directions**: Further studies are required to determine the prevalence and mechanisms underlying vascular and neurodegenerative pathology in iCAA.

## 3 RESULTS

The baseline demographic and clinical characteristics of the cohort are provided in Table 1; the mean age at presentation was 42 years, and six of 11 patients (55%) were male. Nine of 11 (82%) patients could be classified as “probable” iCAA and two of 11 (18%) as “possible” iCAA using the modified Queen Square diagnostic criteria.^3^, ^4^ Ten (91%) had childhood procedures with confirmed use of cadaveric dura; the original operative notes for the remaining patient were unavailable; however, the use of cadaveric dura was highly likely as this was routine for durotomy procedures at the treating hospital during this period. Genetic testing was performed in nine patients for causative variants associated with familial AD and CAA in APP (including copy number variants), PSEN1 and PSEN2 genes; no relevant variants were identified. One patient had a history of childhood learning disability but was functionally independent in adulthood.

**TABLE 1:** Summary of clinical features.

Patient	Sex	Procedure	Use of cadaveric dura	Age at exposure (years)	Age at symptom onset (years)	Latency (years)	Duration of follow‐up (years)	Presenting symptom	Alive at paper finalization
1^*^	M	Posterior fossa papilloma resection, open surgery	Yes	11	48	37	8	Generalized seizures	Yes
2^*^	M	Parotid hemangioma embolization	Yes	0	27	27	9	ICH	Yes
3^*^	F	Open repair of left parietal skull fracture	Yes	0	34	34	9	ICH	No
4	M	Embolization of right posterior auricular AVM	Yes	9	38	29	6	ICH	Yes
5^*^	F	Right facial cavernous hemangioma embolization	Yes	5	43	38	3	TFNE	No
6	F	Lumbar laminectomy for diastematomyelia and tethered cord repair, open surgery	Yes	3	40	37	5	Migraine	Yes
7	M	Lumbar laminectomy for spina bifida repair, open surgery	Yes	0	40	40	5	TFNE	Yes
8	M	Right frontotemporal meningioma removal, open surgery	Unknown	13	54	41	4	ICH	Yes
9	F	Lumbar laminectomy for conus ependymoma removal, open surgery	Yes	15	50	35	4	ICH	Yes
10	F	Lumbar laminectomy for spina bifida repair, open surgery	Yes	11	52	41	2	Migraine	Yes
11	M	Open repair of left parietal skull fracture	Yes	5	36	31	3	ICH	Yes

Abbreviations: AVM, arterio‐venous malformation; CAA, cerebral amyloid angiopathy; ICH, intracerebral hemorrhage; TFNE, transient focal neurological episodes.

* Details for Cases 1, 2, 3, and 5 have been published previously.^3^, ^13^

At presentation (ICH, *n* = 6; transient focal neurological episodes [TFNE], *n* = 2; migraine, *n* = 2; seizures, *n* = 1; Table 1), no cognitive concerns were reported. At the time of the first assessment in our center, no evidence of MTA was observed in any of the patients. One patient demonstrated Koedam and global cortical atrophy (GCA) scores of 1, most likely attributable to previous childhood surgery (Table 2).

**TABLE 2:** Summary of key investigation findings.

Patient	Change in atrophy scores between baseline and follow‐up MRI (point change)			Cerebral microbleeds	Cortical superficial siderosis	ICH (number of events)	CSF markers	Amyloid PET	Histopathology	Genetic testing
	MTA	Koedam	GCA							
1	+2	+1	+1	Multiple	Disseminated	1	Not performed	Not performed	Biopsy, CAA	Negative
2	+1	0	0	Multiple	Disseminated	9	Aβ‐42 low, total tau/Aβ‐42 normal	Deposition in all regions of the cerebral cortex	Biopsy, CAA	Negative
3	0	0	0	Multiple	Focal	3	Aβ 42 low, Total Tau/Aβ‐42 normal	Deposition in all regions of the cerebral cortex	Not performed	Negative
4	+1	0	0	Multiple	No	1	Not performed	Not performed	Biopsy, CAA, Alzheimer's (Aβ, tau)	Negative
5	+2	+1	+1	Multiple	Disseminated	3	Not performed	Not performed	Full *post mortem* examination, CAA, Alzheimer's (Aβ, tau)	Negative
6	0	+1	0	Multiple	Disseminated	0	Not performed	No uptake in the cortical regions	Not performed	Negative
7	+1	+1	+1	Multiple	Disseminated	0	Not performed	Deposition in all regions of the cerebral cortex	Not performed	Negative
8	0	+1	+1	Multiple	Disseminated	1	Not performed	Not performed	Not performed	Not performed
9	+3	+2	+2	Multiple	Disseminated	2	Not performed	Not performed	Not performed	Not performed
10	+1	0	+1	Multiple	Disseminated	0	Not performed	Deposition in all regions of the cerebral cortex	Not performed	Negative
11	0	0	0	Multiple	Disseminated	6	Aβ 42 low, Total Tau/Aβ‐42 normal	Not performed	Not performed	Negative

Abbreviations: Aβ, amyloid beta; CAA, cerebral amyloid angiopathy; CSF, cerebrospinal fluid; GCA, global cortical atrophy score (0 to 3); ICH, intracerebral hemorrhage; MTA, medial temporal atrophy score (0 to 4).

The median duration of follow‐up was 5 years (interquartile range [IQR]: 3.5 to 6.5 years). Within the follow‐up period, recurrent ICH occurred in eight of 11 patients, with four patients experiencing multiple events. All patients had at least one formal neuropsychometric assessment, with a median interval of 3 years between their initial presentation and their most recent evaluation (IQR: 2 to 4 years) (Table 3). Seven patients met criteria for a minor cognitive disorder, two for a major cognitive disorder, including one of the participants with multiple recurrent ICH. Six patients had impairment in three or more cognitive domains. Impaired performance was most commonly observed for tests of verbal IQ (*n* = 8), performance IQ (*n* = 7), and processing speed (*n* = 6).

**TABLE 3 alz71640-tbl-0003:** Summary of key cognitive assessment findings.

Patient	Time from presentation to most recent cognitive assessment (years)	Learning disability	Level of education	Age at onset of cognitive symptoms (years)	Minor/major/no cognitive disorder at follow‐up	Number of domains impaired	Cognitive impairment pattern at most recent assessment
1	8	Yes	School	45	Minor	3	Verbal IQ, processing speed, and EF
2	3	No	University	39	Major	3	Language, visuoperception, and EF
3	2	No	University	n/a	None	1	Verbal IQ
4	3	No	University	31	Minor	2	Performance IQ, processing speed,
5	2	No	University	n/a	none	0	none
6	5	No	University	39	minor	2	Verbal IQ, performance IQ
7	5	No	School	50	minor	5	Verbal IQ, performance IQ, naming, processing speed, EF
8	2	No	University	45	minor	4	Verbal IQ, performance IQ, processing speed, EF
9	3	No	University	37	major	3	Verbal IQ, performance IQ, memory and processing speed
10	2	No	University	46	minor	2	Verbal IQ, performance IQ
11	3	No	University	42	minor	3	Verbal IQ, performance IQ, processing speed

Abbreviations: EF, executive functions; IQ, intelligence quotient.

**FIGURE 1:**
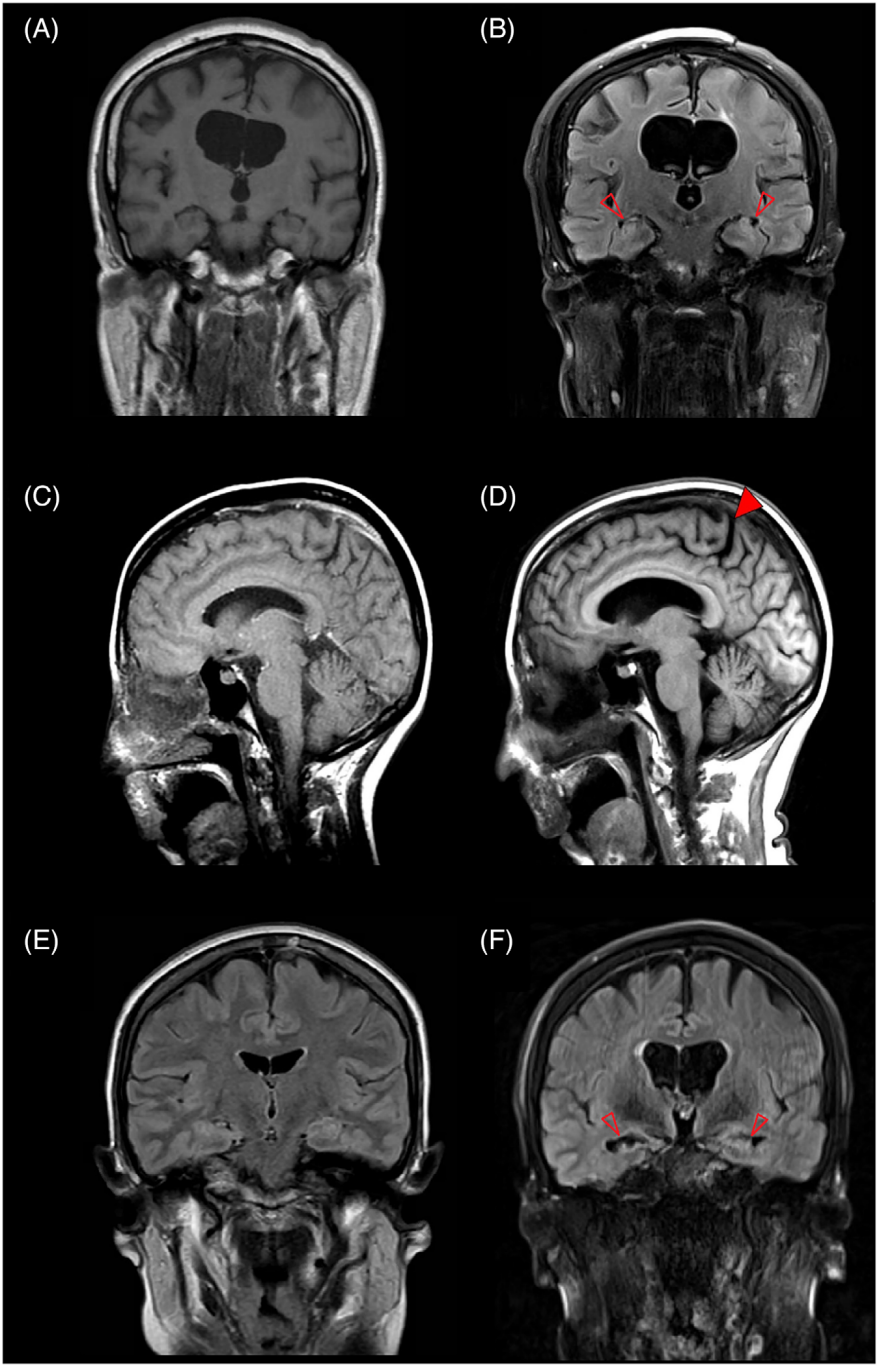
Examples of neuroimaging changes in reported patients. (A and B) Patient 1. Sequential images (A, T1‐weighted; B, T2‐weighted fluid‐attenuated inversion recovery [FLAIR], 4 years later) show progressive hippocampal atrophy (empty red arrowheads). (C and D) Patient 5. FLAIR images 18 months apart show progression of parietal atrophy, most apparent in the pars marginalis (red arrow). These changes are contralateral to the hematoma and therefore unlikely to relate to this. (E and F) Patient 9. At presentation, there is no evidence of regional or generalized atrophy on coronal FLAIR images (E). Repeat imaging 2 years later (F) shows extensive atrophy, particularly affecting the hippocampi and medial temporal lobes (empty red arrowheads).

All patients underwent an initial MRI scan at the time of clinical presentation and had at least one follow‐up scan (median interval: 3 years, IQR: 2 to 6.5 years). Compared with baseline imaging, eight patients demonstrated progression in atrophy, as reflected by increased MTA, Koedam, or GCA scores. Three patients had scores consistent with moderate to severe atrophy at follow‐up (score ≥2; Table 2). Representative MRI images are provided in Figure 1A–F.

Histopathological examination was performed in four patients – three through biopsy and one via *post mortem* examination. In addition to CAA histopathology observed in all examined patients, AD neuropathological change, characterized by diffuse parenchymal Aβ deposits, a meshwork of neuropil threads, pretangles, occasional neurofibrillary tangles, and sporadic Aβ‐ and tau‐positive neuritic plaques, was confirmed in two cases.

A summary of the clinical, radiological, and pathological data is presented in Table S2.

## 4 DISCUSSION

In a cohort of 11 patients with iCAA, we found that nearly three‐quarters developed imaging evidence of progressive brain atrophy over a median follow‐up of 5 years. From a cognitive perspective, although none reported impairments at baseline, over a median follow‐up of 3 years, neuropsychological testing revealed deficits in the majority (9/11, 82%), with a predominantly “vascular” subcortical phenotype. Some patients had biomarkers, histopathological changes, or both, suggestive of Alzheimer's co‐pathology, suggesting that this might be of relevance for disease progression in certain patients with iCAA. Our observations suggest that individuals with iCAA, despite their relatively young age at presentation in comparison to typical “sporadic” CAA, may be at increased risk of developing neurodegeneration and subsequent cognitive impairment.

Two major questions arise from our observations. The first is whether cognitive and neurodegenerative changes in patients with iCAA arise purely from “vascular” factors, that is, by the combination of repeated episodes of intracranial hemorrhage and associated cerebral damage consequent to dysfunction of cortical and leptomeningeal cerebral small vessels affected by Aβ,^14^ or whether these patients, like many with sporadic CAA, also have co‐existing Alzheimer's pathology. Establishing the answer to this question is not straightforward, particularly if, as seems likely, both vascular and Alzheimer's pathologies contribute to the phenotypes observed. A pattern of cognitive deficits that is cortical (typically amnestic, but allowing for other phenotypes including language, dysexecutive, and posterior cortical presentations) rather than subcortical might support a primary role for Alzheimer's disease, although these patterns are non‐specific and detailed data on the domain‐specific cognitive deficits in CAA‐related cognitive impairment remain limited.^8^, ^15^, ^16^ In particular, studies examining cognition in iCAA are scarce; a recent cross‐sectional study (*n* = 20) reported subtle executive dysfunction associated with poorer perceived general health and physical functioning but did not include follow‐up data.^17^

Aβ biomarkers in routine clinical use, such as cerebrospinal fluid (CSF) Aβ‐42 and amyloid positron emission tomography (PET), cannot reliably distinguish between Alzheimer's disease and CAA; CSF Aβ‐40, which is more discriminative, is not widely used outside of research settings.^18^ Pathological aggregation of tau protein is a defining histopathological feature of Alzheimer's disease and might be a better discriminator in this setting; measurement of CSF tau is widely available, and other biomarkers (tau PET, plasma phosphorylated tau at threonine 217 (p‐tau217)) can help diagnose Alzheimer's disease and monitor disease progression and, hence, could be of interest for future studies in iCAA.^19^, ^20^ In our cohort, brain tissue examination was performed in four patients, confirming tau pathology consistent with Alzheimer's disease neuropathological change in two cases. CSF biomarkers were assessed in three patients, none of whom exhibited elevated CSF tau levels. These findings suggest that co‐existing Alzheimer's disease–related pathology may be present in some patients with iCAA.^21^

A second question to consider – whether patients with iCAA can indeed have co‐existing Alzheimer's type tau pathology – is whether this develops consequent to Aβ pathology or is the result of independent tau transmission during their original medical procedural exposure. Compelling genetic and biomarker data support Aβ as the root cause of AD, with Aβ aggregation as the initial step and trigger in AD pathogenesis, with other factors (such as tau deposition) later driving the complex neurodegenerative process.^22^ Tau can be seeded in rodent models independently of Aβ,^23^ and although there are no reports to date of human‐to‐human tau transmission independent of Aβ, histopathological data from iatrogenic Aβ cases suggest that tau pathology might only develop after longer incubation times.^21^ Cadaveric pituitary‐derived human growth hormone contains measurable quantities of tau,^2^ but equivalent data for cadaveric dura mater are lacking.[Table alz71640-tbl-0003]

We acknowledge limitations. Our data were derived from a small cohort who had assessments and investigations during their routine clinical care; consequently, there is some variability in the data available for each person. However, iCAA remains rare, so despite these unavoidable limitations, we believe our observations have relevance for other clinicians involved in caring for patients with iCAA.

We encourage vigilance for signs of cognitive impairment in patients with iCAA and recommend investigations including neuropsychometry, imaging, and assessment of neurodegenerative biomarkers to allow timely recognition and appropriate further management.

## AUTHOR CONTRIBUTIONS

**LP, GB**, and **DJW**: Conception and design of the work. **LP** and **SFH**: Collection of clinical data. **LP, GB, DM, RO**, and **DJW**: Analysis and interpretation of the clinical data. **LP** and **GB**: Drafting of the manuscript. **DJW** and **DM**: Contribution to the clinical‐radiological diagnosis. **DM**: Figure preparation. **EC**: Neuropsychological data acquisition, analysis, and interpretation. **ZJ**: Neuropathological examination, analysis, and interpretation of data. **VH, RO**, and **DJW**: Contribution to the management and follow‐up of the presented cases. **DJW, GB**, and **JC**: Revision of the manuscript. All authors read and approved the final manuscript.

## CONFLICTS OF INTERESTS STATEMENT

David Werring receives funding as a NIHR Senior Investigator and has received grant funding from the Stroke Association, British Heart Foundation, Rosetrees Trust, Innovate UK, University College London Hospitals (UCLH) Biomedical Research Centre, and National Brain Appeal Innovations fund; speaking honoraria from Bayer; speaking and chairing honoraria from AstraZeneca (Alexion) and NovoNordisk; and consultancy fees from Alnylam, Bayer, and NovoNordisk. He was Chief Investigator for the PROHIBIT‐ICH and OPTIMAS trials and is UK Co‐Chief Investigator for the ELAPSE and DO‐IT trials. David has participated on the data safety monitoring board for OXHARP and the LACI‐2, TICH‐2, TICH‐3, RESTART, MACE‐ICH and PLINTH Trial Steering Committees. The other authors report no competing interests. Author disclosures are available in the Supporting Information.

## FUNDING INFORMATION

No funding was received for this work.

## CONSENT STATEMENT

Patients or their next of kin gave written consent for publication.

## Supporting information

Supporting Information

Supporting Information

## Data Availability

The data that support the findings of this study are available from the corresponding author upon reasonable request. Data are not publicly accessible due to privacy restrictions concerning sensitive information of research participants.
