# Supplementary material for: Evidence for progressive neurodegeneration in iatrogenic cerebral amyloid angiopathy
Source: Alzheimers Dement. 2026 Jun 30;22(7):e71640. doi: 10.1002/alz.71640 (PMC13319396; doi:10.1002/alz.71640)
Supplement: Supplementary file 1 — Supporting Information [file ALZ-22-e71640-s002.docx]

**Supplementary material.**

**Supplementary Table 1. List of Neuropsychological Tests Administered**

| Premorbid Intellectual functioning |
| --- |
| National Adult Reading Test (NART)^1^ |
| General intellectual functioning |
| Wechsler Adult Intelligence Scale – 3^rd^ Edition (WAIS-III)^2^ |
| Raven Advanced Progressive Matrices – Set 1^3^ |
| Memory |
| Recognition Memory Tests (RMT), Words and Faces^4^ |
| Adult Memory and Information Processing Battery (AMIPB), Story and Figure recall^5^ |
| The Camden Memory Test: Topographical Recognition Memory Test^6^ |
| The Camden Memory Test: Paired Associate Learning Test^6^ |
| The Doors and People Test^7^ |
| Naming |
| Graded Naming Test^8^ |
| Oldfield Naming Test^9^ |
| Visuo-perception |
| Visual Object and Space Perception Battery (VOSP)^10^ |
| Executive functions |
| Stroop Colour Word Test^11^ |
| Hayling Sentence Completion Test^12^ |
| Modified Card Sorting Test^13^ |
| Cognitive Estimation Test^14^ |
| Phonemic fluency^15^ |
| Speed of Processing |
| Symbol Digit Modalities Test (SDMT)^16^ |
| Trail-Making Test Part A^17^ |
| ‘A’ Cancellation^18^ |

**References to List of Neuropsychological Tests Administered**

1. Nelson, & Willison, J. (1991). National adult reading test (NART) : test manual / Hazel E. Nelson with Jonathan Willison (Part II). (2nd edition). NFER-Nelson.
2. Wechsler, D. (1997). WAIS-­‐III administration and scoring manual. The Psychological Corporation, San Antonio, TX
3. Raven, J. C., Court, J. H., Raven, J. C. (John C. ., & Court, J. H. (John H. (1992). Manual for Raven’s progressive matrices and vocabulary scales / by J. Raven, J.C. Raven and J.H. Court.H.K.Lewis & col. ltd.
4. Warrington E. (1984). Recognition Memory Test. Windsor: Nfer-Nelson.
5. Coughlan AK, Hollows SE. The adult memory and information processing battery: The manual. Leeds: Coughlan, 1985.
6. Warrington. (1996). The Camden memory tests / Elizabeth K. Warrington. Psychology Press.
7. Baddeley, A. D., Emslie, H., & Nimmo-Smith, I. (2006). Doors and people: A test of visual and verbal recall and recognition. Harcourt Assessment.
8. Warrington, E. K. (1997). The Graded Naming Test: A Restandardisation. Neuropsychological Rehabilitation, 7(2), 143–146. <http://doi.org/10.1080/713755528>
9. Oldfield RC, Wingfield A. Response latencies in naming objects. Q J Exp Psychol 1965;17:273–81.
10. Warrington EK, James M. Visual Object and Space Perception Battery. Bury St.
11. Trenerry, M. R., Crosson, B., DeBoe, J., & Leber, W. R. (1989). Stroop neuropsychological screening test. Odessa, FL: Psychological Assessment Resources.
12. Burgess, P. W., & Shallice, T. (1997). The Hayling and Brixton tests. Technical Report, Thames Valley Test Company, Bury St. Edmunds (UK), 1997
13. Nelson, H. E. (1976). A modified card sorting test sensitive to frontal lobe defects. Cortex, 12(4), 313-324.
14. MacPherson, Wagner, G. P., Murphy, P., Bozzali, M., Cipolotti, L., & Shallice, T. (2014). Bringing the cognitive estimation task into the 21st century: normative data on two new parallel forms.  PloS One, 9(3), e92554–e92554.

https://doi.org/10.1371/journal.pone.0092554

1. Tombaugh, Kozak, J., & Rees, L. (1999). Normative Data Stratified by Age and Education for Two Measures of Verbal Fluency: FAS and Animal Naming. Archives of Clinical Neuropsychology, 14(2), 167–177. https://doi.org/10.1016/S0887-6177(97)00095-4
2. Smith, A. (1982). Symbol digit modalities test (p. 22). Los Angeles, CA: Western Psychological Services.
3. Army Individual Test Battery (1944). Manual of directions and scoring. Washington, DC: War Department, Adjutant General’s Office.
4. Willison JR, Warrington EK. Cognitive retardation in a patient with preservation of psychomotor speed. Behav Neurol 1992;5:113–6.

**Supplementary Table 2. Summary of clinical, radiological and pathological data**

| **Demographic** | |
| --- | --- |
| Number of patients | 11 |
| Age at presentation, years, mean (SD) | 42 (8.3) |
| Male sex, n (%) | 6 (55%) |
| Duration of follow-up, years, median (IQR) | 5.0 (3.5 to 6.5) |
| Known learning disability, n (%) | 1 (9%) |
| University level of education, n (%) | 9 (82%) |
| **Imaging** | |
| Time of most recent imaging relative to first presentation, years, median (IQR) | 3 (2 to 6.5) |
| MTA on latest imaging, median grade (IQR) | 1 (0 to 1.5) |
| Koedam score on latest imaging, median grade (IQR) | 1 (0 to 1) |
| GCA on latest imaging, median grade (IQR) | 1 (0 to 1) |
|  |  |
| **Neuropsychology** | |
| Time of most recent assessment relative to first presentation, years, median (IQR) | 3 (2 to 4) |
| Minor cognitive disorder, n (%) | 6 (55%) |
| Major cognitive disorder, n (%) | 2 (18%) |
| Number of domains impaired per patient, median (IQR) | 2 (1.5 to 3) |
| Domain-specific impairments, n (%) | 25 (100%) |
| Verbal IQ | 7 (28%) |
| Performance IQ | 7 (28%) |
| Processing speed | 5 (20%) |
| Executive function | 3 (12%) |
| Memory | 1 (4%) |
| Naming | 1 (4%) |
| Visuoperception | 1 (4%) |
|  |  |
| **CSF biomarkers** (n=3) | |
| Reduced Aβ 1-42, n (%) | 3 (100%) |
| Elevated total/phosphorylated Tau-181, n (%) | 0 |
|  |  |
| **Histopathology** (n=4) | |
| CAA severity, grade, median (IQR) | 3 (2.75 to 3) |
| Parenchymal Aβ deposition, n (%) | 3 (75%) |
| Tau pathology, presence, n (%) | 2 (50%) |

***Abbreviation: Aβ, amyloid beta; CAA, cerebral amyloid angiopathy; CSF, cerebrospinal fluid; GCA, global cortical atrophy score (0-3); IQ, intelligence quotient; MTA, medial temporal atrophy score (0-4).***
